# Perceptual inference is impaired in individuals with ASD and intact in individuals who have lost the autism diagnosis

**DOI:** 10.1038/s41598-020-72896-6

**Published:** 2020-10-13

**Authors:** Sagi Jaffe-Dax, Inge-Marie Eigsti

**Affiliations:** 1 Department of Psychology, Princeton University, Princeton, NJ 08544 USA; 2 Department of Psychological Sciences and Connecticut Institute for the Brain and Cognitive Sciences, University of Connecticut, Storrs, CT 06269 USA

**Keywords:** Perception, Language, Human behaviour

## Abstract

Beyond the symptoms which characterize their diagnoses, individuals with autism spectrum disorder (ASD) show enhanced performance in simple perceptual discrimination tasks. Often attributed to superior sensory sensitivities, enhanced performance may also reflect a weaker bias towards previously perceived stimuli. This study probes perceptual inference in a group of individuals who have lost the autism diagnosis (LAD); that is, they were diagnosed with ASD in early childhood but have no current ASD symptoms. Groups of LAD, current ASD, and typically developing (TD) participants completed an auditory discrimination task. Individuals with TD showed a bias towards previously perceived stimuli—a perceptual process called “contraction bias”; that is, their representation of a given tone was contracted towards the preceding trial stimulus in a manner that is Bayesian optimal. Similarly, individuals in the LAD group showed a contraction bias. In contrast, individuals with current ASD showed a weaker contraction bias, suggesting reduced perceptual inferencing. These findings suggest that changes that characterize LAD extend beyond the social and communicative symptoms of ASD, impacting perceptual domains. Measuring perceptual processing earlier in development in ASD will tap the causality between changes in perceptual and symptomatological domains. Further, the characterization of perceptual inference could reveal meaningful individual differences in complex high-level behaviors.

## Introduction

Autism Spectrum Disorder (ASD) is characterized by impairments in social communication and by the presence of repetitive, perseverative or stereotyped behaviors^1^. Beyond core diagnostic criteria, individuals with ASD also exhibit distinct perceptual aptitudes. For example, in the auditory domain, people with ASD display an enhanced ability to distinguish the pitch of pairs of simple tones^2^ and the pitch of pairs of spoken word and nonword speech^3^. In addition to fine-grained processing of the auditory signal, these tasks also require the listener to make perceptual inferences about stored representations of sounds. That is, the listener compares a stored representation to the current observation. Following the logic of Bayesian inference, the sounds presented in a given trial (*t*) are perceived as more similar to prior stimuli (*t*−1) than if they were presented in isolation; this perceptual change is described as a “contraction” towards the stored representation^4–7^. Depending on the task, reduced perceptual inferencing (and specifically contraction) may either enhance or, more often, impede performance on perceptual discrimination tasks^8,9^. The current study utilized an auditory “Same-Different” task, in which perceptual inferencing impedes performance, to test whether impaired Bayesian inference could underlie the frequently-reported auditory perceptual advantages characteristic of ASD.

### Loss of diagnosis

While ASD was originally considered a lifelong disorder, research indicates that between 8 and 20% of individuals with ASD will present with no symptoms by the time they reach adolescence^10^. A growing literature has documented many aspects of the loss of autism diagnosis (LAD; formerly, “optimal outcome”) phenomenon, in which LAD individuals are largely indistinguishable from their typically developing (TD) peers, with both groups differing from individuals who remain on the spectrum. Studies have examined behavior using standardized clinical assessments of social and communication skills^11^, restricted and repetitive behaviors^12^, psychiatric comorbidities^13^, language and verbal memory^14^, executive functions^15^, and academic skills^16^. Across each of these studies, participants in the LAD group scored in the average range or higher, with performance similar to (or higher than) that of the TD group. Experimental behavioral studies have revealed similar performance in LAD and TD groups for ratings of likeability and broader autism phenotype^17^, and for a variety of pragmatic language abilities^18–21^. This behavioral work provides a foundation for further exploration of the range of possible outcomes (including questions about how to define an optimal outcome^22^), and the nature of the neural systems that support such sharp changes in developmental trajectories^23^.

While it is clear that clinically meaningful improvements in social and communication skills, and the absence of repetitive, stereotyped and perseverative behaviors and interests, are observed in a significant subgroup of individuals with ASD, there are more open questions than answers. One of the exciting unstudied issues centers on the strengths that characterize ASD^24^. The diagnosis is associated with a remarkable set of perceptual and cognitive strengths in visuospatial processing^25^, musical skills^26^, solving puzzles^27^, etc. The current paper asks whether perceptual strengths in LAD are effectively normalized, or whether they are retained even when the clinical symptoms of the disorder have remitted. In the absence of longitudinal data, it is not certain that individuals with the LAD outcome originally displayed perceptual strengths.

Sequential discrimination tasks provide a sensitive means of evaluating how representations of stimuli are impacted by prior information—a process often termed perceptual Bayesian inference. For example, in two-tone pitch discrimination tasks, a participant encounters a long sequence of trials containing similar stimuli from a single category (i.e., pure tones), and is asked to make a same/different judgment about each pair. The representation of the first stimulus in each trial is noisier, or less robust, than the representation of the second (more recent) stimulus, at the time of the response^28^. To compensate for this degradation, findings suggest that the representation of the first stimulus is *merged with prior representations*; thus, at the point of comparison, the perceived difference between its representation and the second stimulus differs from the physical difference between the stimuli. The level of divergence between the inferred perception and the physical stimuli, as measured by accuracy judgments, can inform us about the degree to which an individual relies implicitly on prior information^29^. Performance thus also provides an index of how much an individual relies on the most recent versus prior stimuli^30^.

Recent research on ASD has probed inferencing in perceptual discrimination tasks. Some studies indicate a weaker reliance on the most recent stimuli^31–34^ (but see ref.^35^). For example, in a two-tone frequency discrimination task, high-functioning adults with ASD showed a reduced contraction of the perceived stimulus towards their representation of the most recent item^36,37^.

The current paper presents a novel re-analysis of previously-described data^38^ to examine how ASD, and more specifically, LAD, is associated with perceptual inference. A prior study examining d’ in this task indicated heightened (better) pitch discrimination in ASD; in contrast, the LAD group’s abilities did not differ from those of TD controls, but showed an intermediate pattern of performance between the ASD and the TD groups^38^. The current study employs a perceptual inference analysis (as described in ref.^30^) to test whether individuals who no longer have an ASD diagnosis will maintain ASD-like reduced perceptual inferencing, or will present with perceptual processes that look more like those of TD peers. Given their previously reported poorer overall performance^38^ we predicted that the LAD group would have more contraction towards recently-presented stimuli than that of the ASD group. A greater contraction could account for their observed poorer performance. The current manuscript provides a novel evaluation of whether the loss of ASD diagnosis entails a more typical pattern of perceptual inferencing; it also sheds further light on global statistical learning of stimulus priors in ASD.

## Methods

Individuals with LAD (*n* = 27), ASD (*n* = 29), and TD (*n* = 23) were assessed in a same-different two-tone discrimination task. All procedures were approved by the University of Connecticut Institutional Review Board and were in accordance with the Declaration of Helsinki. Informed consent was obtained from the participant or legal guardian prior to the study. Groups did not significantly differ on full-scale IQ and chronological age. Participants ranged in age from 8 to 21 years; all had cognitive abilities in the normal range. Additional details are shown in Table 1; see also refs.^38,39^. In each of 120 trials, two 100 ms tones were presented, and participants were asked to indicate whether the two tones were “same” or “different”. The first tone was randomly chosen to be 500 Hz, 750 Hz, 1000 Hz, or 1500 Hz. The second was of the same frequency (“same” trials; 50%) or a frequency of 1%, 2% or 3% above the first tone (“different trials”; 50%). The inter-tone interval was 1 s, and participants had unlimited time to respond; the inter-trial interval was 500 ms. The experiment included 120 trials across three blocks of 40 trials per block (20 same and 20 different). Blocks were ordered by increasing difficulty (decreasing frequency difference in the “different” trials), with first tone frequency presented in random order. The first block contained trials that differed by 3% of total frequency, the second by 2%, and the third by 1%. Prior to task administration, participants completed a short training block with feedback (16 trials total; 8 at 4% and 8 at 1% frequency difference levels). Training was repeated until participants reached an accuracy level of 75% (no participant required more than 16 trials of training). Trial data and analysis scripts are available from the authors on request.

**Table 1:** Demographic information for ASD, Loss of ASD Diagnosis (LAD), and typically developing (TD) groups.

	ASD *M* (*SD*)	LAD *M* (*SD*)	TD *M* (*SD*)	*χ*^2^ or *F*	*p*	*η*^*2*^_*p*_
*N* (M:F)	29 (25:4)	27 (21:6)	23 (17:6)	0.93	0.63	
Chron. age (yrs)	12.3 (2.3)	12.5 (3.6)	13.7 (2.9)	1.37	0.26	0.002
	8–17	8–21	9–21			
Nonverbal IQ^a^	111 (14)	112 (14)	115 (12)	0.60	0.55	0.002
	78–147	92–142	89–139			
Verbal IQ^a^	104 (13)*	113 (13)	113 (12)	4.91	0.01	0.16
	81–133	91–137	99–136			
Fullscale IQ^a^	109 (13)	116 (12)	116 (11)	2.97	0.06	0.07
	80–138	96–139	101–142			
ADOS Com + Soc^b^	10.3 (3.0)*	1.7 (2.1)	0.8 (1.1)	155.48	< .001	0.82
	7–19	0–5	0–4			
ADOS Repetitive^c^	1.2 (1.1)	0.4 (0.6)	0 (0)	10.8	< 0.001	0.3
	0–3	0–2	0–0			
SCQ Total (*Lifetime*)^d^	23.0 (5.9)*	16.5 (6.6)*	1.4 (1.3)*	91.57	< .001	0.24
	10–33	5–28	0–4			
Age of first words	21.0 (11.2)	26.9 (11.6)		3.32	0.08	0.10
(months)	6–54	8–48				

Asterisks identify means that differ significantly from other means in the comparison not sharing that superscript. Data are presented as *M(SD),* range.

^a^Wechsler Abbreviated Scale of Intelligence (WASI^47^) Nonverbal, Verbal, and Fullscale IQ.

^b^Autism Diagnostic Observation Schedule^48^. *Communication plus social domain summed score.* Cutoff is 7 for ASD and 10 for autistic disorder.

^c^Restrictive and repetitive behaviors domain score.

^d^Social Communication Questionnaire^49^; for *Lifetime* scale, 15 is the ASD cutoff.

**Figure 1:**
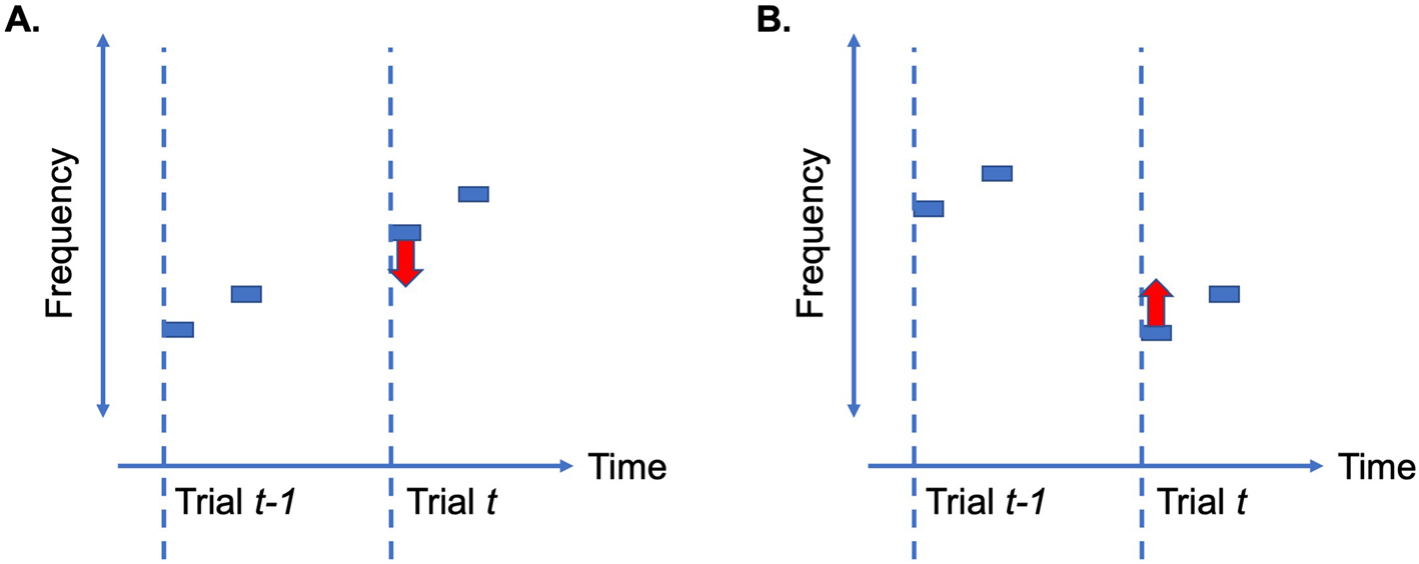
Schematic illustration of trial types. (**A**) Example of *Bias* + trial. The tones are denoted by blue rectangles. The beginning of each trial is represented by dashed lines. The first tone in the current trial (trial *t*) is flanked by the second tone and the tones in the previous trial (trial *t* − 1). The perceptual representation of the first tone is degraded compared to the representation of the second tone and is more contracted towards previously perceived stimuli (denoted by the red arrow). In the *Bias* + trials, this contraction increases the perceived difference between the two tones in the current trial and eases the identification of these trials as “different”. (**B**) Example of *Bias*- trial. The second tone in the current trial (trial *t*) is flanked by the first tone in the current trial and the tones in the previous trial (trial *t* − 1). Perceptual contraction of the first tone towards the tones in the previous trial decreases the perceived difference between the tones in the current trial and increases the chances of incorrectly identifying this trial as “same”.

We analyzed each participant’s response as a function of the tones presented in the preceding trial (*t* − 1). Analyzing this impact captures a *recency* effect, where, based on previous work^37^, individuals with ASD should differ from TD individuals. Given the smaller number of trials in the current work, we could not evaluate the separate contribution of all previous trials in addition to the contribution of the most recent trial. We contrasted performance in two trial types. In ***Bias + trials***, the pitch of the initial tone in the current trial (*t*) is flanked by the pitch of the tones of pair *t* − 1 (the previous stimulus) and the pitch of the second tone in pair *t* (example trial in Fig. 1A). Thus, in *Bias* + trials, contraction of the first tone in trial *t* towards the stimulus in trial *t* − 1 **increases** the perceived difference between the two tones in trial *t* (red arrow in Fig. 1A), making it easier to judge the pair as “different.” In ***Bias-trials***, the pitch of the second tone in trial *t* is flanked by the pitch of the tones in trial *t* − 1 and the pitch of the first tone in trial *t* (example trial in Fig. 1B); Thus, in *Bias-* trials, contraction of the first tone toward the previous stimulus **decreases** the perceived difference between the two tones in the current trial (red arrow in Fig. 1B), making it harder to judge the pair as “different.” Trials in which the correct response should be “same” were also included in the *Bias-* group, since contraction of the first tone towards the previous stimulus decreased the chance of making a correct judgment.

We hypothesized that the difference between trial types would be larger for the TD and LAD groups than for the ASD group, reflecting a larger impact of the previous stimulus on current perception individuals among the TD and LAD groups, compared to individuals with ASD. In the psychophysics literature, the perceptual “contraction” of the representation of the first tone of the stimulus pair in trial *t* towards the stimulus presented in trial *t* − 1 is thought to measure sequential dependency^29,40^. This is also thought to reflect a central tendency or summary statistical learning^4,5,8^; that is, global statistical learning of the stimulus prior. In the current perceptual inference task, Bias + trials should induce facilitatory contraction, and Bias- trials should cause inhibitory contraction. Given this operationalization of perceptual inference, the two predictions above led to the following specific hypotheses: (1) Because its members have impaired perceptual inference, the ASD group should perform worse on Bias + trials, and better on Bias- trials, than the TD group, due to reduced facilitatory and inhibitory contraction on these trials. (2) The LAD group should perform better on Bias + trials and worse on Bias- trials than the ASD group due to typical facilitatory and inhibitory contraction on these trials.

Following prior work^36,37^, we included only individuals whose task performance was significantly above chance (> 60% accuracy in the physically easiest condition of 3% frequency difference, as determined by assuming binomial distribution of random responses in these 30 trials). The logic behind this exclusion criterion is that error analysis (such as bias comparison) is only meaningful if there was a cognitive difference between correct and incorrect responses, i.e., when the difference between the tone was above the individual’s limen. Participants who were generally at chance may not have been engaged in the same task; they may simply have been responding at random. Note that if all participants, whose performance was simply above 50% on the easiest condition, were included in the analyses, results were effectively similar.

All analyses of main effects and interactions were conducted using linear mixed-effects models in Matlab R2018b (Mathworks, MA), with group and trial type as fixed variables and subject as a random variable. For specific post-hoc contrasts between two groups, we included only the comparison groups in the model and corrected for multiple comparisons by dividing the significance threshold by the number of parallel comparisons (Bonferroni correction).

## Results

**Figure 2:**
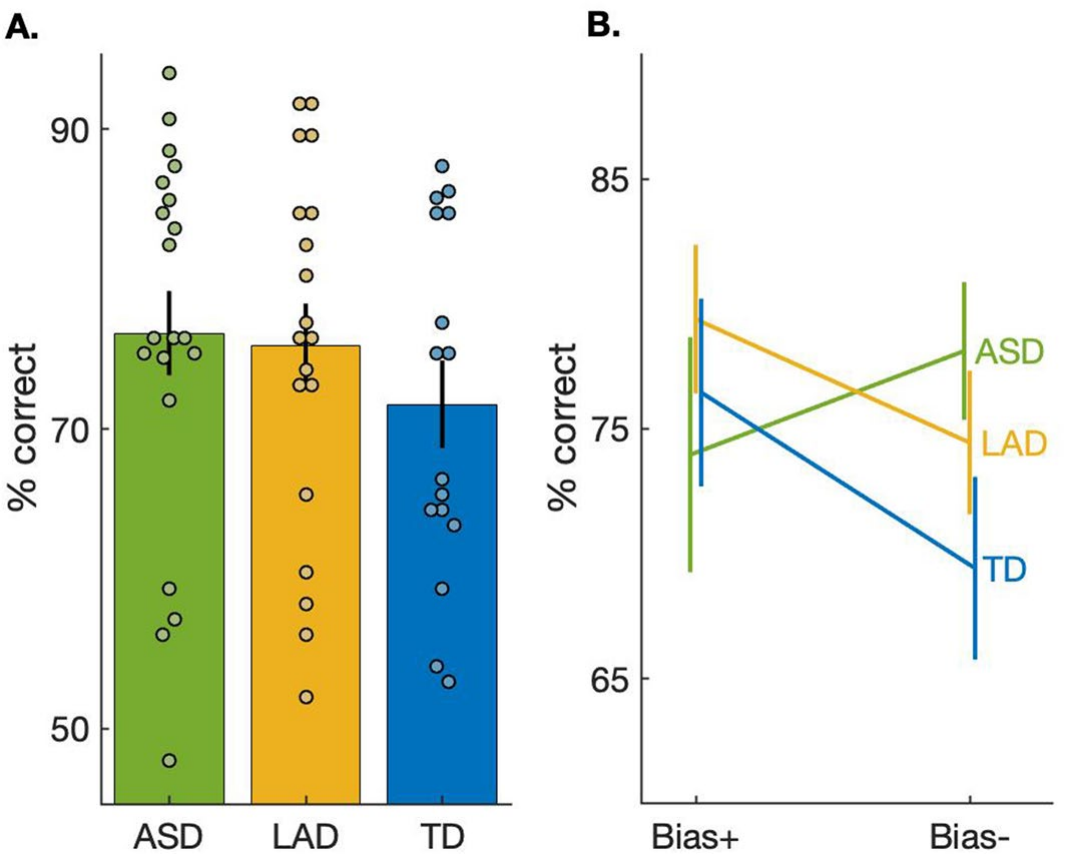
Performance on the two-alternative forced choice frequency discrimination task. (**A**) Overall accuracy by group. We did not find a significant group difference in overall performance. Dots represent individual results. Bars represent group averages. Error bars represent SEM. (**B**) Performance as a function of trial type. Lines represent groups’ averages. Error bar represent SEM. Individuals in LAD and TD groups performed better on Bias + trials, where the consideration of the preceding stimulus improved discrimination, relative to Bias- trials, where the previous stimulus distracts from correct discrimination. Mixed effects model (see Methods) results indicate a significant within group effect of trial type: LAD: *F*(1,1522) = 6.2, *p* = 0.01. TD: *F*(1,1310) = 5.4, *p* = 0.01. These effects remained significant when controlling for multiple comparisons (Bonferroni correction). There was no significant trial type difference for the ASD group, *F*(1,1541) = 0.76, *p* = 0.38.

As reported previously^38^, individuals in the TD group had lower overall accuracy. Twenty-one of 29 from the ASD group (72%), 21 of 27 from the LAD group (78%), and 18 of 23 individuals (78%) from the TD group, were included in all subsequent analyses. This relatively high exclusion rate was expected for participants of this chronological age, for a challenging and fairly tedious task. Groups did not differ on the fraction of participants that were excluded due to chance level performance. The overall accuracy difference between groups was not significant, *F*(2,4374) = 1.8, *p* = 0.17; see Fig. 2A. Individuals with typical development had a slightly faster mean reaction time compared to the ASD and LAD groups (915 ± 45 ms, 952 ± 84 ms and 954 ± 79 ms; Mean ± SEM for TD, ASD and LAD, respectively); however, there was no significant group difference, *F*(2,4374) = 1.1, *p* = 0.32.

The final sample size was powered to detect a medium or large effect (Cohen’s *d* > 0.44 for power of *1* *−* *β* = 0.8). The impact of bias was clearly apparent, given that in the TD group, there was a significant effect of trial type (Bias- versus Bias +) on accuracy, *F*(1,1310) = 5.4, *p* = 0.02, Cohen’s *d* = 0.43. Thus, with our sample size, we had sufficient power to find an effect of trial type.

Turning to the analysis of primary interest—the assessment of perceptual inference—linear mixed effects models suggested no significant main effect on accuracy of either Group, *F*(2,4373) = 0.97, *p* = 0.38, or Bias trial type (*Bias* + vs *Bias-*), *F*(1,4373) = 1.5, *p* = 0.21. Importantly, there was a significant Group X Bias trial type interaction, *F*(2,4373) = 3.02, *p* = 0.048. Specifically, there was a significant difference between LAD and ASD groups, *F*(1,3064) = 19.4, *p* = 0.00001, such that the LAD group exhibited a larger difference in performance than the ASD group between *Bias* + and *Bias-* trials. Similarly, there was a significant difference between the TD and ASD groups in the impact of bias, *F*(1,2852) = 7.6, *p* = 0.006. These effects remained significant when controlling for multiple comparisons (Bonferroni correction). The LAD and TD groups did not significantly differ on the difference between *Bias* + and *Bias-* trials, *F*(1,2833) = 2.2, *p* = 0.14; results are shown in Fig. 2B.

With regards to reaction times, linear mixed effects models suggested no significant main effect of Group, *F*(2,4373) = 0.79, *p* = 0.45, or Bias trial type (*Bias* + vs *Bias-*), *F*(1,4373) = 0.59, *p* = 0.44. There was no significant Group X Trial type interaction, *F*(2,4373) = 0.026, *p* = 0.97. The individual measures reported in Table 1 (age, FSIQ, ADOS and SCQ sores, and age of first words) did not contribute any unique variance to the Group X Trial type interaction, all *p*’s > 0.1, calculated within groups.

Altogether, results show that participants in the LAD and TD groups were more susceptible to the influence of prior stimuli, relative to the ASD group. This finding suggests that individuals with LAD update their perceptual representations according to prior context, while individuals with ASD have a more veridical perceptual representation. Group differences in sensitivity did not appear to be a simple outcome of speeded responding, given the absence of group differences in RT. If anything, faster responders could have shown a reduced sensitivity to the influence of prior stimuli, which is not the case here.

## Discussion

With intensive intervention, some children diagnosed with ASD in early childhood (previously described as having an optimal outcome) later go on to lose the symptoms of this neurodevelopmental disorder. Beyond improvements in communication and social skills, results of the current study suggest that children with a “loss of ASD diagnosis” (LAD) also display typical perceptual inference skills, unlike their peers with ASD. Specifically, similar to typically developing youth, participants in the LAD group were susceptible to the influence of recently-presented stimuli in a sequential discrimination judgement task. This result suggests that individuals in the LAD group update their perceptual representations flexibly and rapidly, in contrast to individuals with ASD.

These findings suggest that weaker perceptual updating of the statistical properties of recent contexts, recently reported for individuals with ASD^37^, is linked to the symptoms of ASD. That is, when an individual loses the ASD diagnosis, they also show a typical ability to update their statistical representation of recent context. In contrast to previous work^37^, we did not have sufficient trials to compare the impact of the most recent trial with the impact of all previously-presented stimuli. In general, the current findings are quite consistent with a growing body of literature examining the hypothesis that ASD reflects impairments in harnessing statistical regularities to make predictions and extract generalizations^31–37^.

Updating one’s representation of an auditory stimulus, according to the influence of a prior stimulus, might permit more nuanced perception of (for example) verbal prosody, or of the differences among phonological features, which are the fundamental units of speech sounds. In contrast, less malleable (less immediately updated) perceptual representations might predict the presence of absolute pitch abilities; indeed, such abilities have been extensively documented in ASD^41–43^. Further research might seek to link these perceptual processes to their distinct physiological bases, on the one hand, and to fine-grained processing and encoding of speech and music stimuli, on the other hand.

## Limitations

The current findings reflect cross-sectional data; they cannot resolve two alternatives. One possibility is that individuals in the LAD group displayed ASD-typical perceptual updating earlier in development, when their ASD symptoms were salient, but that this perceptual “profile” shifted in concert with broader behavioral changes (possibly reflecting an underlying cognitive change). An alternative possibility is that children in the LAD group displayed a TD-typical perceptual profile from early in development, even while they displayed ASD symptoms; although diagnosed with ASD in early childhood, they did not share the characteristic perceptual profile. This might indicate more fundamental differences between individuals who eventually comprise the LAD and TD groups, and would illuminate more basic characteristics of ASD as a disorder.

It is also possible that perceptual updating abilities played a role in the response to speech and language interventions. Given the latter, if perceptual inferencing abilities were identified early in development, we might test whether these skills are associated with a sharper improvement in speech and language skills, given the relevant intervention. Longitudinal designs that better characterize participants early in development, and that assess perceptual inference abilities before and after intervention, are needed to disentangle these alternative explanations. Further research must also consider the impact of factors such as age and IQ.

One interesting point regarding the task design is that participants had unlimited time to respond. Consistent differences in RT would lead to longer intervals since the preceding stimulus as well as the one before it. The decay of one’s representation of a stimulus is expected to be larger for the most recent stimulus, since representations decay exponentially as a function of time^44^. In this study, the longer RTs for the ASD group might have contributed to an overestimation of their contraction bias and therefor an underestimated of group differences. A study design encouraging speeded responding (e.g., by time-out) would likely have revealed even larger group differences that might have reflected meaningful individual correlates with other behavioral measures—a point relevant to any future research.

Our groups differed on the gender ratio (the ASD group had a larger proportion of male participants), which could contribute to the group difference; the sample was not sufficiently powered for testing interactions of our main comparison with gender. In addition, the ASD group showed a larger variance in the impact of bias on perception, suggesting that this group might be a combination of multiple sub-groups with different perceptual characteristics.

Finally, the current study was not designed specifically to test the effects of perceptual priming. Some prior results suggest that, at least in the domain of semantic priming, individuals with ASD show reduced susceptibility to priming^45^, though the relevance of this study to the present is fairly distant. Certainly, the current findings provide an impetus for further research on the nature of priming of all types, and how it may differ in ASD with respect to perceptual processing.

## Conclusions

The current findings suggest that the relation between basic perceptual aptitudes and high-level cognitive communication and social skills may provide an exciting basis from which to better understand individual differences in the development of language and social skills. This relationship has been previously demonstrated in the context of dyslexia, the most prevalent learning disability. In a study of dyslexia that used similar experimental methods, reading skill acquisition was related to the ability to compensate for noisy observation by integrating prior knowledge^46^. Findings in the current study support the possibility that perceptual inference—the ability to weight current observation against prior contextual information—is where social cognition meets perception.

## Data Availability

The datasets during and/or analyzed during the current study available from the corresponding author on reasonable request.

## Abbreviations

ASD: Autism spectrum disorder
LAD: Loss of autism diagnosis
TD: Typical development
